# The interplay between math anxiety and working memory on math performance: a longitudinal study

**DOI:** 10.1111/nyas.14722

**Published:** 2021-12-24

**Authors:** Sandra Pellizzoni, Elisa Cargnelutti, Alessandro Cuder, Maria Chiara Passolunghi

**Affiliations:** ^1^ Department of Life Sciences University of Trieste Trieste Italy; ^2^ Dipartimento/Unità Operativa Pasian di Prato Scientific Institute, IRCCS E. Medea Udine Italy

**Keywords:** math learning, math anxiety, working memory, cognitive factors, developmental trajectories

## Abstract

Mathematical skills are essential to mastering everyday activities, making professional choices, and exercising citizenship in a numerate society. There is extensive evidence of the relationship between math anxiety (MA) and working memory (WM) influencing math attainment. Studies have mainly considered adult samples, however, leaving primary school children almost unexplored. This study is a first attempt to examine how the complex interplay between MA and WM affects math achievement from a developmental perspective. A total of 148 third graders were assessed with WM, general anxiety (GA), MA, and math tasks. Anxiety and WM were assessed at the beginning of the school year when children started attending grade 3, while math achievement was tested twice at the start of grades 3 and 4. The findings seem to confirm that GA has both a direct and an indirect effect (mediated by WM) on math performance in third and fourth graders. MA has a direct effect on math performance in grade 4, but only an indirect effect in grade 3, suggesting MA has a developmental trajectory, becoming stronger over time. The implications in the educational setting are discussed, pointing to the importance of a combined intervention on MA and WM.

## Graphical abstract

Here, we examine how the complex interplay between math anxiety (MA) and working memory (WM) affects math achievement from a developmental perspective. One hundred forty‐eight third graders were assessed for WM, general anxiety (GA), MA, and math tasks. MA and WM were assessed at the beginning of grade 3, while math achievement was tested twice at the start of grades 3 and 4. GA has both a direct and an indirect effect on math performance in third and fourth graders. MA has a direct effect on math performance in grade 4 but only an indirect effect in grade 3, suggesting MA has a developmental trajectory becoming stronger over time.

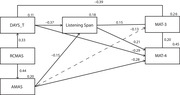

## Introduction

Numbers are an essential part of our lives and daily activities (in cooking, shopping, managing money, and reading the clock). Numerical abilities assessed at an early age predict crucial life factors, such as academic success,^1^ employment opportunities,^2^, ^3^ salary size,^4^ socioeconomic status,^5^, ^6^ and personal and social well‐being,^6^, ^7^ and they are fundamental to an informed and active citizenship.

Given the importance of numerical abilities, it is crucial to elucidate the factors that can promote or hinder the process involved in learning this school subject. The literature on the topic has extensively investigated the general cognitive abilities required, with working memory (WM) emerging as one of the most important factors for academic success.^8^, ^9^ In addition to such general cognitive abilities, emotional factors also seem to have a role in math attainment, and math anxiety (MA) has been the object of in‐depth studies over the last 60 years.^10^, ^11^, ^12^ Being aware of the importance of cognitive and other factors to math attainment, researchers are now focusing on their ability to predict math achievement^13^, ^14^, ^15^ and the influence of their complex interaction on learning.^16^, ^17^, ^18^ That said, only a few contributions to date have focused on how WM and emotional factors mutually affect math proficiency, especially in younger students.^19^, ^20^, ^21^, ^22^

This study is thus one of the first attempts to conduct a longitudinal study on the interplay between one of most robust cognitive math precursors (WM) and relevant emotional factors (general anxiety (GA) and MA) and to examine their specific contribution to math achievement with reliable tools. The aim is to extend the knowledge gained from previous work on this theme.^19^

### Working memory and math abilities

WM is a limited‐capacity system that enables information to be stored temporarily and manipulated.^23^, ^24^ Multiple learning processes rely on WM. One of the best‐known theorizations of WM is the tripartite model in which there is a central executive responsible for data storage, processing, and monitoring, and two other modality‐dependent systems devoted to processing verbal or visuospatial information.^25^ Although researchers have proposed alternative models to explain how WM functions (such as modality‐independent^26^ or ‐dependent^27^ models), studies in developmental psychology indicate that the tripartite model can explain it best.^8^, ^28^

WM has a well‐established effect on a variety of math domains, such as geometry,^29^, ^30^ mental addition and subtraction,^31^, ^32^ and problem solving.^18^, ^33^ It is well‐known that children with a poor WM are also weak in mathematics.^34^, ^35^, ^36^, ^37^ Recent studies have shown that the relative contributions of memory components (verbal linguistic and nonverbal visuospatial) to general mathematic learning change as children grow older.^38^ When learning and remembering arithmetic, preschoolers seem to rely on visuospatial memory more than on verbal memory.^39^, ^40^ Later, in primary school, learning depends more on verbal rehearsal to store information in memory and, therefore, engages the phonological loop.^41^, ^42^ This change seems to be due to verbally mediated strategies: children start to use verbal code to label symbols and numbers.^43^, ^44^ On the basis of the study on primary school children by Soltanlou *et al*.,^45^ and preliminary correlational analysis of our data, we decided to focus specifically on verbal WM, which seems to be used more extensively when third and fourth graders experience and practice with math tasks.

### General anxiety and math anxiety

Anxiety is defined as a “dispositional and dysfunctional response to a situation perceived as threatening.”^46^ At school, 10% of children experience this condition, which can be seen already in kindergarten.^47^ High levels of anxiety have been observed in children with learning difficulties or disabilities, who are typically described as more anxious than their classmates.^48^ While the detrimental effect of emotional factors, such as anxiety, on children has been acknowledged, their influence on children's academic performance has been underexplored, in particular, if compared with the literature that focused on the cognitive abilities.^9^, ^49^, ^50^

How to measure GA in young children remains a critical issue. Self‐assessments are scarcely reliable, probably due to the complexity of the construct. Teachers’ assessments have proved a better indicator of children's emotional states (e.g., Refs. 51, 52, 53) and could also predict their math achievement (e.g., Ref. 19). Teachers’ ratings can, therefore, be taken as a useful measure of children's anxiety.^54^

If this dysfunctional response is aroused by a particular stimulus, then we can speak of a particular type of anxiety. MA is “a feeling of tension and anxiety that interferes with the manipulation of numbers and the solving of mathematical problems in ordinary life and academic situations.”^55^ At school, the prevalence of MA is in the range of 2–17%,^55^, ^56^ depending on the student population considered and the criteria used to define the condition.

Recent meta‐analytic investigations confirm a significant negative correlation between MA and mathematics performance (range: −0.30 < *r* < −0.34), and this connection starts to take root early in a child's school career.^57^, ^58^, ^59^ A crucial question in the debate on the emergence of MA concerns whether it is a cause or a consequence of math difficulties. In 77% of cases, children with severe MA have a typical or better mathematics performance,^16^ suggesting that the cognitive and emotional problems relating to mathematics are largely dissociated. However, the mechanisms underlying this relationship between cognitive and emotional factors shaping children's math achievements remain to be clarified.

### The link between WM and MA

As mentioned earlier, there is extensive evidence of the relationship between MA and WM influencing math attainment. A recent meta‐analysis found a moderate negative association between anxiety and WM.^60^ To date, WM has been the factor most often studied with a view to explaining the relationship between MA and math performance. One of the theories advanced to do so is called the processing efficiency theory (PET),^61^ developed from Baddeley's model of WM, which suggests that anxious thoughts (e.g., worries) influence WM by reducing its capacity. Several studies demonstrated that MA had a detrimental effect on math achievement because it reduced the individual's WM resources.^62^, ^63^, ^64^ There are two different hypotheses regarding the type of person who would be more exposed to this effect. Ashcraft and Kirk^62^ claimed that adults more gifted in relation to WM could manage both math tasks and anxiety‐driven thoughts more successfully and would seem to be unaffected by such a connection. An alternative view is that people with a better WM are more likely to experience math difficulties caused by MA, especially when coping with math tasks in more stressful situations (“choking under pressure”).^63^

In undergraduate students experiencing severe MA, a recent fMRI experiment^65^ showed an exaggerated response even to easy math problems across series of trials, and their reaction times were longer. This increase in processing time could be the sign of a greater WM load across all levels of task difficulty,^66^ supporting the PET.

From a developmental perspective, the literature indicate that MA interferes with different WM components. In a sample of 11‐ to 15‐year‐old students, Passolunghi *et al*.^67^ found that children with a better verbal WM exhibited less MA. The relationship between MA and math performance seems to exist even in very young children on applied problems^21^ and mathematical application.^22^ Furthermore, a recent meta‐analysis^57^ found that WM mediated the relationship between MA and mathematics. Particularly, the authors found that this relationship did not change according to the WM type or the degree of cognitive control required by the WM task. Some studies examined the role of WM in the relationship between trait anxiety (not MA) and math performance using a mediation approach.^68^, ^69^, ^70^, ^71^ Justicia‐Galiano *et al*.^20^ recently investigated the role of verbal WM and math self‐concept as possible mechanisms mediating between MA and math performance in 8‐ to 12‐year olds. They found that verbal WM mediated the relationship between MA and various math outcomes. This pattern emerged for both teacher‐assessed trait anxiety and children's self‐assessed MA.

### The present study

To date, WM has been the most often studied potential mediator accounting for the relationship between MA and math performance, but data referring to younger students are still particularly scant. We consequently deemed it crucial to further address these themes to (1) investigate both MA and GA (the latter using both self and teacher ratings) using tools with a good reliability index, and to identify their specific contribution to math achievement; (2) examine the developmental link between math performance, cognitive ability (WM), and GA and MA from a longitudinal perspective, in an effort to shed some light on the origins of the link between these factors; and (3) identify a specific developmental trajectory that could connect math with anxiety in a crucial period of a child's schooling. To achieve these goals, we tested primary school children in third and fourth grade—school years that are fundamental both to their math acquisition and to the development of an awareness of their own inner emotional state.^19^

We aimed to extend the results of previous studies in several ways:

1. By further examining MA. Given the importance of how children are assessed on this complex factor,^11^ we used the “Abbreviated Math Anxiety Scale” (AMAS)^72^ in this study. This is a self‐report questionnaire on MA that focuses especially on the emotional aspect of this condition, with good reliability. We wanted to reinforce previous findings^21^ obtained when MA was assessed with a less reliable scale. The AMAS also differs from the scale used by Cargnelutti *et al*.,^19^ so it enabled us to explore the generalizability of previous findings by adopting different assessment tools.
2. By further examining GA. Using both self‐ and teacher‐report questionnaires, and thereby extending previous studies (e.g., Refs. 20 and 22), we investigated whether math performance is influenced by anxiety specific to math, over and above the effect of GA. Unlike Justitia‐Galiano *et al*.,^20^ we compared students’ self‐assessments on GA with teachers’ assessments, considering complex psychological factors. Here again, we used a highly reliable questionnaire for self‐assessed GA that differs from the one chosen by Cargnelutti *et al*.^19^ to see whether their finding of no significant influence of self‐rated anxiety on math achievement was confirmed.
3. By investigating the developmental link between MA and math ability. We considered it crucial to focus on a specific period in children's academic careers, from third to fourth grade. This is when mathematical tasks become more demanding, and any prior negative experiences with math can make children feel anxious about the subject.^54^ Many studies have underscored the importance of considering the association between math performance and anxiety from a developmental perspective.^73^, ^74^, ^75^ Some reports suggest that this association can emerge at some point during primary school and possibly around third grade (e.g., Ref. 54). We consequently followed our students longitudinally up to grade 4.
4. By exploring the interplay between WM and MA, and how it affects math attainment, again from a developmental perspective. This topic has been partially studied in children, drawing on the literature regarding adults and in connection with math acquisition.^21^, ^22^, ^62^ The findings are limited and often contradictory in adult samples (see Refs. 62 and 63), however, making further investigation necessary. To this end, we tested two main assumptions: (1) that WM acts as a mediator between anxiety and math (in order words, anxiety affects WM, which, in turn, affects mathematics); or (2) that anxiety acts as a mediator between WM and math attainment, meaning that the level of WM influences the amount of anxiety, which, in turn, affects math performance. In testing these two alternative hypotheses, we also examined whether these variables can each have a direct effect on math as well.

To reach these goals, children were assessed in two phases. During the first, at the start of their third primary school year, children's cognitive and affective factors were tested, together with their math ability. In the second phase, at the beginning of their fourth year, their math ability was tested again. We used path analysis models to explore the relationships between the variables of interest (i.e., anxiety, WM, and math achievement).

We hypothesized that both GA and MA could have a significant negative effect on math performance. Concerning our two alternative assumptions, we expected the one identifying WM as a mediator between anxiety and math to be the more likely. We also predicted that anxiety would have a direct effect as well as the one mediated by WM. We envisaged a robust relationship between GA and both WM and math performance already at the beginning of grade 3, while we expected the involvement of MA to become stronger over time.

## Methods

### Participants

A total of 158 children in grade 3 were enrolled in the study, but 12 were subsequently excluded for various reasons: five did not obtain their parents’ permission to participate; two had been diagnosed with a specific learning disability; three had a general developmental delay; and two were absent on the day of at least one of the two testing phases. The final sample thus consisted of 146 children (85 females). All participants were Caucasian, came from a middle socioeconomic background (judging from the school records), were native speakers of Italian, and had an average intelligence quotient (as measured with the Vocabulary and Block Design subtests from the WISC‐IV; Italian edition).^76^, ^77^ They were attending 10 different classes at primary schools in northern Italy. At the beginning of the study, children's mean age was 8 years, 4 months (SD = 4). In accordance with the Declaration of Helsinki, a written informed consent form was signed by each child's parents and by the school principals. This study was conducted in compliance with the ethical guidelines of the Italian Association of Psychology and the ethical code of the Italian Register of Professional Psychologists.

### Procedure

Children were tested at two different phases. The first, Time 1 (at the start of grade 3) was devoted to assessing anxiety (children's self‐rated GA and MA and teachers’ ratings of their GA), WM, and math attainment. Then Time 2 (at the start of grade 4) children's math attainment was tested again.

### Tasks

#### WM (verbal WM)

The listening span (LS) task we administered was an Italian adaptation of the test devised by Daneman and Carpenter^78^ used in previous studies (see also Ref. 79). It was chosen as the WM task to include in our model after preliminary analysis showed that it correlated more strongly with the math performance and anxiety measures than other tasks assessing verbal and visuospatial WM (the backward word span, backward digit span, or backward corsi). The task included different levels of difficulty, numbered from 2 to 5 (with Level 2 consisting of two sets of two sentences, Level 3 consisting of two sets of three sentences, and so on), and children were asked to judge the sentences as true or false. Examples of the sentences are: “A and B are the first two letters of the alphabet,” or “The hen is a mammal that lives in the sea.” At the end of each set of sentences, children were asked to recall the last word of each sentence in the order of presentation (“alphabet” and “sea” in the above‐mentioned examples).

#### Anxiety

##### General anxiety

The Revised Children's Manifest Anxiety Scale‒Second Edition (RCMAS‐2; Italian edition)^80^ is a self‐report questionnaire used to identify the source and level of GA in children aged 6–19. We used the short form consisting of 10 items with a simple *yes* (1 point) or *no* (0 points) response format.

The teacher's version of the anxiety subscale of the Depression and Anxiety in Youth Scale (DAYS; Italian edition)^81^ was administered as an additional measure of children's GA (given the previously reported high reliability of teachers’ reports; e.g., Refs. 51, 52, 53 and 81). This subscale consists of seven items with a *yes* (1 point) or *no* (0 points) response format.

##### Math anxiety

The AMAS^72^ is a 9‐item self‐report questionnaire for assessing MA. Using a 5‐point Likert‐type scale (1 = low anxiety to 5 = high anxiety), participants indicated how anxious they would feel during situations involving math.

#### Math abilities

At the beginning of grade 3, we tested children's math performance using the Number module of the standardized MAT‐2 test^82^ developed for children in grade 2 or early in grade 3 (hereafter called MAT‐3), which has a time limit of 20 minutes. The module consists of 11 tasks (e.g., ranking numbers from the smallest to the largest and breaking down composite numbers), each scoring 1 point, if completed correctly.

For the assessment at the beginning of grade 4, we used the same number module in the version developed for children in grade 3 or early in grade 4 (hereafter MAT‐4). This module consists of 13 tasks (e.g., writing down numbers in the range 1–1000 and solving problems involving the concepts of expenses and profits) to be solved within 20 min and each scoring 1 point for correct answers.

## Results

Our data analyses were run using the IBM® SPSS® Statistics 21 software and our path analyses with IBM AMOS. Preliminary analyses revealed no significant differences in math performance across the classes at either of the assessment times: *F*(9,136) = 1.22, *P* = 0.29, partial *η* ^2^ = 0.007, for MAT‐3; *F*(9,136) = 1.79, *P* = 0.08, partial *η* ^2^ = 0.006, for MAT‐4. Descriptive statistics, including task reliability and correlation values between all the tasks, are given in Table 1.

**Table 1:** Descriptive statistics and bivariate zero‐order correlation

		Min	Max	Mean (SD)	Reliability	1	2	3	4	5	6
1	MAT‐3	4.00	11.00	7.82 (1.71)	0.74	–					
2	MAT‐4	1.00	10.00	6.19 (2.08)	0.80	0.47***	–				
3	LS	0.00	4.00	2.21 (.85)	0.86	0.33**	0.45***	–			
4	RCMAS	0.00	6.00	3.97 (1.10)	0.60	−0.20*	−0.32**	−0.20*	–		
5	AMAS	9.00	39.00	20.34 (7.40)	0.90	−0.22**	−0.41***	−0.21*	0.44**	–	
6	DAYS_T	0.00	6.00	1.73 (1.80)	0.66	−0.47***	−0.51***	−0.39***	0.33**	0.15	–

^*^ *P* ≤ 0.05, ^**^ *P* ≤ 0.01, ^***^ *P* ≤ 0.001.

AMAS, Abbreviated Math Anxiety Scale; DAYS_T, the Depression and Anxiety in Youth Scale, assessed by teachers; LS, listening span; Max, maximum; Min, minimum; RCMAS, Revised Children's Manifest Anxiety Scale; SD, standard deviation.

### Models with WM as a mediator between anxiety and math performance

We tested different path analysis models addressing both direct and indirect (mediating) effects by applying a bootstrapping procedure (1000 bootstrap samples). To see which relationships between the variables of interest better explained math performance, we first ran a series of models with the WM measure (i.e., LS) as a mediator. In these models, we tested the different directionality of the link between MAT‐3 and the anxiety measures, but without changing the directionality with MAT‐4 in order to avoid retrospective models. Table 2 shows the statistical fit parameters of these models.

**Table 2:** Statistical fit parameters of the tested models

Model	Description	CMIN	d.f.	CMIN*/*d.f.	*P*	CFI	NFI	TLI	RMSEA	AIC	BCC
WM as a mediator											
1a	AMAS ⟶ MAT‐3 DAYS_T ⟶ MAT‐3	0.95	4	00.07.00	0.99	1.00	1.00	1.07	<0.001	46.30	48.63
1b	MAT‐3 ⟶ AMAS (n.s.) MAT‐3 ⟶ DAYS_T	5.38	4	1.35	0.25	0.99	0.97	0.97	0.05	51.38	53.72
1c	AMAS ⟶ MAT‐3 MAT‐3 ⟶ DAYS_T	2.35	4	0.59	0.67	1.00	0.99	1.03	<0.001	48.35	50.68
1d	MAT‐3 ⟶ AMAS (n.s.) DAYS_T ⟶ MAT‐3	1.13	4	0.28	0.89	1.00	1.00	1.06	<0.001	47.14	49.47
Anxiety as a mediator											
2a	AMAS ⟶ MAT‐3 DAYS_T ⟶ MAT‐3	9.29	5	1.86	0.10	0.98	0.96	0.93	0.08	53.29	55.53
2b	MAT‐3 ⟶ AMAS MAT‐3 ⟶ DAYS_T	12.02	5	2.40	0.04	0.96	0.94	0.89	0.10	56.02	58.25
2c	AMAS ⟶ MAT‐3 MAT‐3 ⟶ DAYS_T	11.33	5	2.27	0.05	0.97	0.95	0.90	0.09	55.37	57.60
2d	MAT‐3 ⟶ AMAS DAYS_T ⟶ MAT‐3	9.22	5	1.84	0.10	0.98	0.96	0.93	0.08	53.22	55.45

AIC, Akaike information criterion; BCC, Browne‐Cudeck criterion

**Figure 1:**
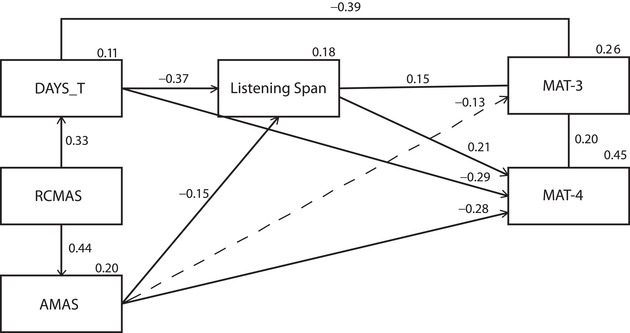
Standardized Model 1a. The dotted line represents a link not surviving the threshold we set for statistical significance (*P = *0.05).

Model 1a (see Fig. 1) had the best statistical fit and a robust theoretical validity, so it was chosen as the best model to compare with the models in which anxiety was the mediator. In this model, the directionality of the link went from anxiety to MAT‐3, as we tested for the effect of both GA and MA on math performance. Both GA assessed by teachers (DAYS_T, *β* = −0.39, *P < 0*.001) and AMAS (*β* = −0.13, *P* = 0.06) were negatively associated with math performance, although the latter association did not survive the threshold we set for statistical significance. LS as well had a significant effect on MAT‐3 (*β* = 0.15, *P* = 0.05) and was also negatively associated with both DAYS_T (*β* = −0.37, *P* < 0.001) and AMAS (*β* = −0.15, *P* = 0.04). As for the remaining relationships, it is worth noting that DAYS_T was also strongly and negatively associated with performance in MAT‐4 (*β* = −0.29, *P <* 0.001), and so was AMAS (*β* = −0.28, *P <* 0.001). The anxiety measures thus predicted not only concurrent, but also future math performance in much the same way as previous math achievement predicted subsequent attainment in this subject. With regard to WM, LS was also associated with performance in MAT‐4 (*β* = −0.21, *P <* 0.01).

### Models with anxiety as a mediator between WM and math performance

In the second series of models, we tested the likelihood of WM (LS) influencing anxiety levels and, as a consequence, the relationship between the latter and math performance (which was only direct in this case). Here again, we examined the different directionality of the relationship between the anxiety measures and MAT‐3, but the link AMAS ⟶ LS was weak and not significant in any of the models (*β* = −0.11, *P* = 0.09), so it was omitted. Table 2 shows the statistical fit indices for the model.

**Figure 2:**
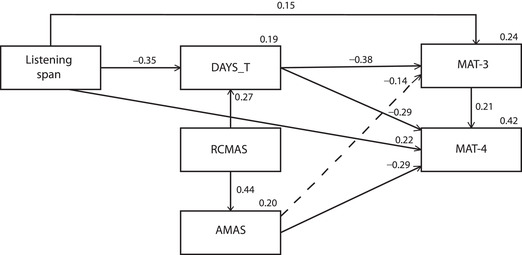
Standardized Model 2a. The dotted line represents a link not surviving the threshold we set for statistical significance (*P = *0.05).

These models generally had a poor statistical fit, the strongest being Model 2a (see Fig. 2), which was used for a comparison with Model 1a. In Model 2a, DAYS_T had a strong negative association with MAT‐3 (*β* = −0.38, *P <* 0.001), while the negative association with AMAS did not survive the threshold we set for statistical significance (*β* = −0.13, *P* = 0.06). LS had a significant association with MAT‐3 (*β* = 0.15, *P* = 0.05), and it was also negatively associated with GA (*β* = −0.35, *P <* 0.001).

### Comparison between the two series of models and description of the model selected

Fit indices for the two series of models that we ran show that the models with anxiety as a mediator had a poor fit, whereas the fit for the models with WM as a mediator ranged from good (Model 1b) to very good (Models 1a, 1c, and 1d). In particular, lower values for AIC, BCC, and RMSEA for the latter more than the former models suggest that it is more reasonable, in statistical terms, too, for anxiety to negatively affect WM than vice versa. The percentage of explained variance of the MAT measures is also slightly higher for Model 1a (*r* ^2^ = 0.26 versus *r* ^2^ = 0.24 for MAT‐3; *r* ^2^ = 0.45 versus *r* ^2^ = 0.42 for MAT‐4), indicating an optimal pattern of relationships between the variables tested in this model for the purpose of explaining math performance. Additional details of Model 1a are given in Table 3.

**Table 3:** Standardized values of Model 1a

Outcome variables	Predictor variables	Direct effects	Scalar estimates	Indirect effects	Total effects	*R* ^2^
MAT‐3	LS RCMAS AMAS DAYS_T	0.15* −0.13 −0.39***	0.076 0.072 0.078	−0.22** −0.02* −0.06*	0.15* −0.22** −0.15* −0.44**	0.26
MAT‐4	MAT‐3 LS RCMAS AMAS DAYS_T	0.20** 0.21** −0.28*** −0.29***	0.150 0.143 0.132 0.152	0.03* −0.30** −0.07** −0.17*	0.20* 0.24* −0.30** −0.35** −0.46**	0.45
LS	RCMAS AMAS DAYS_T	−0.15* −0.37***	0.076 0.076	‐0.19**	−0.19** −0.15* −0.37**	0.54
AMAS	RCMAS	0.44***	0.074		0.44**	0.20
DAYS_T	RCMAS	0.33***	0.078		0.33**	0.11

^*^ *P* ≤ 0.05, ^**^ *P* ≤ 0.01, and ^***^ *P* ≤ 0.001. Significance levels for indirect and total effects correspond to the two‐tailed *P* values derived from the bias‐corrected percentile bootstrapping at 95% CI.

## Discussion

In a numerate and high‐technology world, mathematics rules are fundamental to an individual's personal, educational, and economic success. That is why it is so important to better investigate the complex interplay between emotional and cognitive factors influencing math abilities, both in a prevention and a promotion perspective.^83^ In this study, we aimed to (1) assess MA and GA (from children's and their teachers’ perspective) using tools with a good reliability index, and identify their specific contribution to math performance; (2) investigate the interplay between WM and anxiety (both GA and MA) on math achievement; and (3) follow the developmental trajectory that could connect math performance with GA and MA, in the third and fourth years of primary school.

We first evaluated, across the primary school classes sampled, both statistical significance and effect size of all the possible links between MA and math performance. We concurrently took the impact of GA into account to see whether MA could have a specific role beyond that of GA. We also explored whether anxiety significantly affected math performance directly, even after taking such a strong cognitive math precursor as WM into account. Anxiety and WM were tested at the start of grade 3, whereas math ability was assessed twice, at the start of grades 3 and 4.

We tested several models that differed in the relationship between anxiety and WM, and in their association with math achievement. In the first type of model, WM mediated the relationship between math achievement and anxiety; in the second, anxiety mediated the relationship between math achievement and WM. On comparing the best models (from the statistical and theoretical standpoints) of these two alternative hypotheses, the former—Model 1, with WM as a mediator—was stronger and is discussed below.

It emerged from this model that anxiety had a strong overall impact on math performance at both assessment times, but with important differences. The most relevant measure was children's GA as assessed by their teachers: it negatively affected their concurrent math performance but also predicted that of that assessed a year later, even after accounting for the indirect contribution of previous scores for math achievement. This finding confirms the crucial role of GA in this setting, as already seen in older children (e.g., Ref. 70).

On the other hand, the effect of MA on concurrent math performance in grade 3 did not reach the threshold we set for statistical significance, but MA affected directly and significantly subsequent math performance in early grade 4. This finding seems to confirm, while using different assessment tools, the results of previous studies tracking the onset of a significant link between MA and math performance between grades 3 and 4 (e.g., Refs. 19 and 84). This period could be crucial because (1) the demands of math learning increase and children have to make an effort to keep up; and (2) any prior negative experiences with math learning and achievement may have accumulated enough to undermine their further learning. In short, a vicious cycle can develop, with consequent mutually negative effects on anxiety and performance. Rated at the start of grade 3, MA did not significantly relate to our children's concurrent math performance, but it did predict their performance early in grade 4.

We hypothesize that this earlier lack of a significant relationship between MA and math achievement is attributable not to children's inability to rate their own MA, but to other factors having a more important role at the time. For instance, results of a previous study using latent profile analysis^85^ found that younger students’ MA could be driven by a general tendency toward anxiety, and only older students seem to exhibit more specific forms of anxiety. Similarly, in a study of Mammarella *et al*.^86^ on children attending grades 3–6, the authors found no clear difference between general and academic forms of anxiety. For this reason, it is unlikely that results are influenced by children's inability to assess their own MA, rather results seem to suggest a developmental stage where the boundaries between general and specific anxiety are still vague.

The situation could be different for GA. Our study showed that teachers’ ratings of this variable had a relevant role, whereas children's self‐ratings were not directly related to math achievement at either of the assessment times. Unlike MA, which develops in very specific situations and children can be aware of it from a very early age (e.g., Refs. 21 and 87, 88, 89), GA and its manifestations are less clearly defined and could, therefore, be harder for young children to detect and measure (e.g., Ref. 90). Teachers’ ratings of GA have already proved reliable and useful even for identifying clinically relevant conditions (e.g., Refs. 51, 52, and 91). In line with the above considerations, and even administering a different questionnaire,^72^ children's self‐rated GA was not related to their math performance at the start of grades 3 or 4.

Although previous studies showed that MA has a stronger impact on math performance compared with GA (e.g., Refs. 20, 57, and 86), our results suggest that GA has concurrent (grade 3) and future (early in grade 4) effects on mathematical performance. The results anyway indicate that the most relevant measure was children's GA as assessed by their teachers, whereas children's self‐ratings were not directly related to math achievement at either of the assessment times, confirming previous studies. We believe that, as observed in other studies,^19^ the teacher's rate could synthesize a risk factor that may contribute to the development of a more specific form of anxiety, MA, and, therefore, be indirectly related to mathematical performance that, at this developmental stage, is not captured by self‐evaluation in younger students.

The second aim of our study was to clarify aspects of the role of WM in predicting math performance, and especially its link to anxiety. A single measure of verbal WM capacity (LS) was found positively and directly related to math learning at the beginning of grades 3 and 4. This result confirms the fundamental role of verbal WM as a math precursor (see meta‐analysis in Ref. 92). On the other hand, it came as a surprise when our preliminary analyses revealed no significant impact of the visuospatial component of WM (not included in our path model), as this contradicts previous robust findings (e.g., Refs. 33, 93, and 94). A possible explanation may lie in the type of the math test we used, which is comprehensive of various math skills, but could demand little visuospatial WM processes. Our findings can also be interpreted from a developmental perspective, in that the contribution of the various WM components may differ at different ages, depending on the skills learned in a given developmental stage (see meta‐analysis in Ref. 95).

Our third aim focused on the relationship between WM and anxiety. Here again, it was the teachers’ ratings of GA that showed an association with WM. GA was found to undermine performance in a WM task, in line with previous reports of a detrimental effect of anxiety on WM. It also emerged that WM mediated the indirect association between teachers’ ratings of GA and concurrent and future math performance; in other words, math attainment was also negatively affected by a decrease in WM resources caused by anxiety (e.g., Ref. 66). It is noteworthy that WM also mediated the indirect relationship between MA and both concurrent and future math achievement.

This study has some limitations. First of all, it is necessary to underline that, in path analyses, the definition of the effect directionality can be questionable, and it should be theoretically established rather than statistically provided. Furthermore, the small sample size prevented us from testing more complex models that included additional variables.

Second, it is crucial to note that reliability for DAYS_T, specifically referred to the anxiety scale observed in the students, is not particularly high. Future studies are necessary to replicate our findings with more reliable tools.

Third, a broader evaluation on different forms of negative attitudes to learning (anxiety or depression) and personal assets, such as self‐evaluation and ego resilience,^96^, ^97^ are needed to better understand how the co‐occurrence of a negative attitude and a positive approach may shape the learning process. Linked to this aspect, different questionnaires to measure GA, test anxiety, and MA are needed in an effort to shed more light on the reliability of both self and observer ratings on younger students.

Furthermore, in order to overcome the limits associated with self‐report questionnaires on the developmental sample, future studies should use neurophysiological measures and implicit tasks. Such a comprehensive approach would be needed across all school years to look for any developmental changes in the predictive power of the relationship between anxiety and both cognitive precursors and math performance. Longitudinal models should also be used to investigate a possible feedback effect, with a worse math performance causing more anxiety in a vicious cycle (e.g., Ref. 98).

## Conclusion

The interest of the findings of the present longitudinal study lies in that they show a combined effect of emotional and cognitive factors in predicting both concurrent and future math achievement. They suggest a crucial influence of anxiety as a variable that can consistently impair math attainment. GA was found to have an impact from a very early age, when it also undermined WM, whereas the role of MA appeared to emerge later on.

The findings of this study have important implications in the educational setting. They underscore the teacher's essential role in assessing the emotional complexities of the learning process. The data suggest that children with math difficulties can benefit from early intervention to help them contain and cope with their related anxiety. Such intervention can be run in parallel with more specific math training, as rehabilitation programs that focus only on improving math skills and their cognitive precursors might be ineffective if children do not learn how to handle their negative emotional states at the same time.^99^

## Competing interests

The authors declare no competing interests.

## Author contributions

S.P. and A.C. contributed to the study's conception and design, data acquisition and interpretation, participated in drafting the manuscript, and approved the final version as submitted. E.C. contributed to the study's conception and design, data analysis and interpretation; participated in drafting the manuscript; and approved the final version as submitted. M.C.P. contributed to the study's conception and design, and data interpretation; reviewed its intellectual content; and approved the final version of the manuscript as submitted. All the authors, S.P., A.C., E.C., and M.C.P., take responsibility for the integrity of the data analyzed.

## References

[1] Odic, D. , J.V. Lisboa , R. Eisinger , *et al*. 2016. Approximate number and approximate time discrimination each correlate with school math abilities in young children. Acta Psychol. 163: 17–26. 10.1016/j.actpsy.2015.10.010 26587963

[2] Bynner, J. 1997. Basic skills in adolescents’ occupational preparation. Career Dev. Q. 45: 305–321.

[3] Rivera‐Batiz, F.L. 1992. Quantitative literacy and the likelihood of employment among young adults in the United States. J. Hum. Resour. 27: 313–328.

[4] Dougherty, C. 2003. Numeracy, literacy and earnings: evidence from the National Longitudinal Survey of Youth. Econ. Educ. Rev. 22: 511–521.

[5] Gerardi, K. , L. Goette & S. Meier . 2013. Numerical ability predicts mortgage default. Proc. Natl. Acad. Sci. USA 110: 11267–11271. 23798401 10.1073/pnas.1220568110 PMC3710828

[6] Gross, J. , C. Hudson & D. Price . 2009. The Long‐Term Costs of Numeracy Difficulties. Every Child a Chance Trust and KPMG. East Sussex, UK: National Numeracy.

[7] Furlong, M. , F. McLoughlin , S. McGilloway , *et al*. 2015. Interventions to improve mathematical performance for children with mathematical learning difficulties (MLD). Cochrane Db. Syst. Rev. 4: 1–35.

[8] Gathercole, S.E. , S.J. Pickering , B. Ambridge & H. Wearing . 2004. The structure of working memory from 4 to 15 years of age. Dev. Psychol. 40: 177–190. 14979759 10.1037/0012-1649.40.2.177

[9] St Clair‐Thompson, H.L. & S.E. Gathercole . 2006. Executive functions and achievements in school: shifting, updating, inhibition, and working memory. Q. J. Exp. Psychol. 59: 745–759. 10.1080/17470210500162854 16707360

[10] Carey, E. , F. Hill , A. Devine & D. Szücs . 2016. The chicken or the egg? The direction of the relationship between mathematics anxiety and mathematics performance. Front. Psychol. 6: 1–6. 10.3389/fpsyg.2015.01987 PMC4703847 26779093

[11] Dowker, A. , A. Sarkar & C.Y. Looi . 2016. Mathematics anxiety: what have we learned in 60 years? Front. Psychol. 7: 1–16. 27199789 10.3389/fpsyg.2016.00508 PMC4842756

[12] Peng, P. , T. Wang , C. Wang & X. Lin . 2019. A meta‐analysis on the relation between fluid intelligence and reading/mathematics: effects of tasks, age, and social economics status. Psychol. Bull. 145: 189–236. 30652909 10.1037/bul0000182

[13] Fonteyne, L. , W. Duyck & F. De Fruyt . 2017. Program‐specific prediction of academic achievement on the basis of cognitive and non‐cognitive factors. Learn. Individ. Differ. 56: 34–48.

[14] Higbee, J.L. & P.V. Thomas . 1999. Affective and cognitive factors related to mathematics achievement. J. Dev. Educ. 23: 8–15.

[15] Lu, L. , H.S. Weber , F.M. Spinath & J. Shi . 2011. Predicting school achievement from cognitive and non‐cognitive variables in a Chinese sample of elementary school children. Intelligence 39: 130–140.

[16] Devine, A. , F. Hill , E. Carey & D. Szucs . 2018. Cognitive and emotional math problems largely dissociate: prevalence of developmental dyscalculia and mathematics anxiety. J. Edu. Psychol. 110: 431–444.

[17] Lee, K. , F. Ning & H.C. Goh . 2014. Interaction between cognitive and non‐cognitive factors: the influences of academic goal orientation and working memory on mathematical performance. Educ. Psychol. 34: 73–91.

[18] Passolunghi, M.C. , E. Cargnelutti & S. Pellizzoni . 2019. The relation between cognitive and emotional factors and arithmetic problem‐solving. Educ. Stud. Math. 100: 271–290.

[19] Cargnelutti, E. , C. Tomasetto & M.C. Passolunghi . 2017. The interplay between affective and cognitive factors in shaping early proficiency in mathematics. Trends Neurosci. Educ. 8: 28–36.

[20] Justicia‐Galiano, M.J. , M.E. Martín‐ Puga , R. Linares & S. Pelegrina . 2017. Math anxiety and math performance in children: the mediating roles of working memory and math self‐concept. Br. J. Educ. Psychol. 87: 573–589. 28561304 10.1111/bjep.12165

[21] Ramirez, G. , E.A. Gunderson , S.C. Levine & S.L. Beilock . 2013. Math anxiety, working memory, and math achievement in early elementary school. J. Cogn. Dev. 14: 187–202.

[22] Vukovic, R.K. , M.J. Kieffer , S.P. Bailey & R.R. Harari . 2013. Mathematics anxiety in young children: concurrent and longitudinal associations with mathematical performance. Contemp. Educ. Psychol. 38: 1–10.

[23] Baddeley, A. 1996. The fractionation of working memory. Proc. Natl. Acad. Sci. USA 93: 13468–13472. 8942958 10.1073/pnas.93.24.13468 PMC33632

[24] Baddeley, A. 2003. Working memory: looking back and looking forward. Nat. Rev. Neurosci. 4: 829–839. 14523382 10.1038/nrn1201

[25] Baddeley, A. & G. Hitch . 1974. Working memory. Psychol. Learn. Motiv. 8: 47–89.

[26] Kane, M.J. , S.W. Tuholski , D.Z. Hambrick , *et al*. 2004. The generality of working memory capacity: a latent‐variable approach to verbal and visuospatial memory span and reasoning. J. Exp. Psychol. Gen. 133: 189–217. 15149250 10.1037/0096-3445.133.2.189

[27] Shah, P. & A. Miyake . 1996. The separability of working memory resources for spatial thinking and language processing: an individual differences approach. J. Exp. Psychol. Gen. 125: 4–27. 8851737 10.1037//0096-3445.125.1.4

[28] Giofrè, D. , E. Borella & I.C. Mammarella . 2017. The relationship between intelligence, working memory, academic self‐esteem, and academic achievement. J. Cogn. Psychol. 29: 731–747.

[29] Giofrè, D. , I.C. Mammarella & C. Cornoldi . 2014. The relationship among geometry, working memory, and intelligence in children. J. Exp. Child Psychol. 123: 112–128. 24709286 10.1016/j.jecp.2014.01.002

[30] Giofrè, D. , I.C. Mammarella , L. Ronconi & C. Cornoldi . 2013. Visuospatial working memory in intuitive geometry, and in academic achievement in geometry. Learn. Individ. Differ. 23: 114–122.

[31] Caviola, S. , I.C. Mammarella , D. Lucangeli & C. Cornoldi . 2014. Working memory and domain‐specific precursors predicting success in learning written subtraction problems. Learn. Individ. Differ. 36: 92–100.

[32] Mammarella, I.C. , S. Caviola , C. Cornoldi & D. Lucangeli . 2013. Mental additions and verbal‐domain interference in children with developmental dyscalculia. Res. Dev. Disabil. 34: 2845–2855. 23810925 10.1016/j.ridd.2013.05.044

[33] Passolunghi, M.C. & I.C. Mammarella . 2010. Spatial and visual working memory ability in children with difficulties in arithmetic word problem solving. Eur. J. Cogn. Psychol. 22: 944–963.

[34] Hitch, G.J. & E. McAuley . 1991. Working memory in children with specific arithmetical learning difficulties. Br. J. Psychol. 82: 375–386. 1954527 10.1111/j.2044-8295.1991.tb02406.x

[35] Passolunghi, M.C. & F. Pazzaglia . 2005. A comparison of updating processes in children good or poor in arithmetic word problem‐solving. Learn. Individ. Differ. 15: 257–269.

[36] Passolunghi, M.C. & L.S. Siegel . 2004. Working memory and access to numerical information in children with disability in mathematics. J. Exp. Child Psychol. 88: 348–367. 15265681 10.1016/j.jecp.2004.04.002

[37] Siegel, L.S. & E.B. Ryan . 1989. The development of working memory in normally achieving and subtypes of learning disabled children. Child Dev. 60: 973–980. 2758890 10.1111/j.1467-8624.1989.tb03528.x

[38] Giofrè, D. , E. Donolato & I.C. Mammarella . 2018. The differential role of verbal and visuospatial working memory in mathematics and reading. Trends Neurosci. Educ. 12: 1–6.

[39] McKenzie, B. , R. Bull & C. Gray . 2003. The effects of phonological and visual‐spatial interference on children's arithmetical performance. Educ. Child Psychol. 20: 93–108.

[40] Simmons, F. , C. Singleton & J. Horne . 2008. Brief report—phonological awareness and visual‐spatial sketchpad functioning predict early arithmetic attainment: evidence from a longitudinal study. Eur. J. Cogn. Psychol. 20: 711–722.

[41] Hitch, G.J. , S. Halliday , A.M. Schaafstal & J.M.C. Schraagen . 1988. Visual working memory in young children. Mem. Cogn. 16: 120–132. 10.3758/bf03213479 3352517

[42] Rasmussen, C. & J. Bisanz . 2005. Representation and working memory in early arithmetic. J. Exp. Child Psychol. 91: 137–157. 15890174 10.1016/j.jecp.2005.01.004

[43] Geary, D.C. , C.C. Bow‐Thomas , F. Liu & R.S. Siegler . 1996. Development of arithmetical competencies in Chinese and American children: influence of age, language, and schooling. Child Dev. 67: 2022–2044. 9022227

[44] Logie, R.H. , K.J. Gilhooly & V. Wynn . 1994. Counting on working memory in arithmetic problem solving. Mem. Cogn. 22: 395–410. 10.3758/bf03200866 7934946

[45] Soltanlou, M. , S. Pixner & H.C. Nuerk . 2015. Contribution of working memory in multiplication fact network in children may shift from verbal to visuo‐spatial: a longitudinal investigation. Front. Psychol. 6: 1–10. 26257701 10.3389/fpsyg.2015.01062 PMC4512035

[46] Lewis, A. 1970. The ambiguous word “anxiety”. Int. J. Psychiatry 9: 62–79. 4921639

[47] Egger, H.L. & A. Angold . 2006. Common emotional and behavioral disorders in preschool children: presentation, nosology, and epidemiology. J. Child Psychol. Psychiatry 47: 313–337. 16492262 10.1111/j.1469-7610.2006.01618.x

[48] Fisher, B.L. , R. Allen & G. Kose . 1996. The relationship between anxiety and problem‐solving skills in children with and without learning disabilities. J. Learn. Disabil. 29: 439–446. 8763559 10.1177/002221949602900412

[49] Alloway, T.P. & M.C. Passolunghi . 2011. The relationship between working memory, IQ, and mathematical skills in children. Learn. Individ. Differ. 21: 133–137.

[50] Rohde, T.E. & L.A. Thompson . 2007. Predicting academic achievement with cognitive ability. Intelligence 35: 83–92.

[51] Kendall, P.C. , A.C. Puliafico , A.J. Barmish , *et al*. 2007. Assessing anxiety with the child behavior checklist and the teacher report form. J. Anxiety Disord. 21: 1004–1015. 17270388 10.1016/j.janxdis.2006.10.012

[52] Lyneham, H.J. , A.K. Street , M.J. Abbott & R.M. Rapee . 2008. Psychometric properties of the School Anxiety Scale ‐Teacher report (SAS‐TR). J. Anxiety Disord. 22: 292–300. 17339095 10.1016/j.janxdis.2007.02.001

[53] Salbach‐Andrae, H. , K. Lenz & U. Lehmkuhl . 2009. Patterns of agreement among parent, teacher and youth ratings in a referred sample. Eur. Psychiatry 24: 345–351. 18789656 10.1016/j.eurpsy.2008.07.008

[54] Cargnelutti, E. , C. Tomasetto & M.C. Passolunghi . 2017. How is anxiety related to math performance in young students? A longitudinal study of grade 2 to grade 3 children. Cogn. Emot. 31: 755–764. 26935005 10.1080/02699931.2016.1147421

[55] Richardson, F.C. & R.M. Suinn . 1972. The mathematics anxiety rating scale: psychometric data. J. Couns. Psychol. 19: 551–554.

[56] Chinn, S. 2009. Mathematics anxiety in secondary students in England. Dyslexia 15: 61–68. 19089884 10.1002/dys.381

[57] Caviola, S. , E. Toffalini , D. Giofrè , *et al*. 2021. Math performance and academic anxiety forms, from sociodemographic to cognitive aspects: a meta‐analysis on 906,311 participants. Educ. Psychol. Rev. 10.1007/s10648-021-09618-5.

[58] Namkung, J.M. , P. Peng & X. Lin . 2019. The relation between mathematics anxiety and mathematics performance among school‐aged students: a meta‐analysis. Rev. Educ. Res. 89: 459–496.

[59] Zhang, J. , N. Zhao & Q.P. Kong . 2019. The relationship between math anxiety and math performance: a meta‐analytic investigation. Front. Psychol. 10: 1–17. 31447719 10.3389/fpsyg.2019.01613 PMC6692457

[60] Moran, T.P. 2016. Anxiety and working memory capacity: a meta‐analysis and narrative review. Psychol. Bull. 142: 831–964. 26963369 10.1037/bul0000051

[61] Eysenck, M.W. & M.G. Calvo . 1992. Anxiety and performance: the processing efficiency theory. Cogn. Emot. 6: 409–434.

[62] Ashcraft, M.H. & E.P. Kirk . 2001. The relationships among working memory, math anxiety, and performance. J. Exp. Psychol. Gen. 130: 224–237. 11409101 10.1037//0096-3445.130.2.224

[63] Beilock, S.L. & T.H. Carr . 2005. When high‐powered people fail: working memory and “choking under pressure” in math. Psychol. Sci. 16: 101–105. 15686575 10.1111/j.0956-7976.2005.00789.x

[64] Young, C.B. , S.S. Wu & V. Menon . 2012. The neurodevelopmental basis of math anxiety. Psychol. Sci. 23: 492–501. 22434239 10.1177/0956797611429134 PMC3462591

[65] Pizzie, R.G. , N. Raman & D.J. Kraemer . 2020. Math anxiety and executive function: neural influences of task switching on arithmetic processing. Cogn. Affect. Behav. Neurosc. 20: 309–325. 10.3758/s13415-020-00770-z 32112298

[66] Beilock, S.L. 2008. Math performance in stressful situations. Curr. Dir. Psychol. Sci. 17: 339–343.

[67] Passolunghi, M.C. , S. Caviola , R. De Agostini , *et al*. 2016. Mathematics anxiety, working memory, and mathematics performance in secondary‐school children. Front. Psychol. 7: 1–8. 26869951 10.3389/fpsyg.2016.00042 PMC4735424

[68] Ganley, C.M. & M. Vasilyeva . 2014. The role of anxiety and working memory in gender differences in mathematics. J. Educ. Psychol. 106: 105–120.

[69] Ng, E. & K. Lee . 2015. Effects of trait test anxiety and state anxiety on children's working memory task performance. Learn. Individ. Differ. 40: 141–148.

[70] Owens, M. , J. Stevenson , J.A. Hadwin & R. Norgate . 2012. Anxiety and depression in academic performance: an exploration of the mediating factors of worry and working memory. School Psychol. Int. 33: 433–449.

[71] Owens, M. , J. Stevenson , R. Norgate & J.A. Hadwin . 2008. Processing efficiency theory in children: working memory as a mediator between trait anxiety and academic performance. Anxiety Stress Coping 21: 417–430. 18686056 10.1080/10615800701847823

[72] Hopko, D.R. , R. Mahadevan , R.L. Bare & M.K. Hunt . 2003. The Abbreviated Math Anxiety Scale (AMAS) construction, validity, and reliability. Assessment 10: 178–182. 12801189 10.1177/1073191103010002008

[73] Dowker, A. 2005. Early identification and intervention for students with mathematics difficulties. J. Learn. Disabil. 38: 324–332. 16122064 10.1177/00222194050380040801

[74] Ma, X. & N. Kishor . 1997. Assessing the relationship between attitude toward mathematics and achievement in mathematics: a meta‐analysis. J. Res. Math. Educ. 28: 26–47.

[75] Mata, M.D.L. , V. Monteiro & F. Peixoto . 2012. Attitudes towards mathematics: effects of individual, motivational, and social support factors. Child Dev. Res. 10.1155/2012/876028.

[76] Wechsler, D. 2003. Wechsler Intelligence Scale for Children. San Antonio, TX: Psychological Corporation.

[77] Orsini, A. & L. Pezzuti . 2012. WISC‐IV: Contributo alla taratura italiana. Florence, Italy: Giunti OS.

[78] Daneman, M. & P.A. Carpenter . 1980. Individual differences in working memory and reading. J. Verbal Learn. Verbal Behav. 19: 450–466.

[79] Passolunghi, M.C. , C. Cornoldi & S. De Liberto . 1999. Working memory and intrusions of irrelevant information in a group of specific poor problem solvers. Mem. Cogn. 27: 779–790. 10.3758/bf03198531 10540807

[80] Reynolds, C.R. , B.O. Richmond , F. Sella , *et al*. 2012. *Revised Children's Manifest Anxiety Scale*. 2nd ed. Florence, Italy: Giunti OS.

[81] Newcomer, P.L. , E.M. Barenbaum & B.R. Bryant . 1995. Depression and Anxiety in Youth Scale. Austin, TX: PRO‐ED.

[82] Amoretti, G. , L. Bazzini , A. Pesci & M. Reggiani . 2007. MAT‐2. Test di matematica. Florence, Italy: Giunti O.S.

[83] Pellizzoni, S. , G.M. Apuzzo , C. De Vita , *et al*. 2020. Exploring EFs and math abilities in highly deprived contexts. Front. Psychol. 11: 1–7. 32210893 10.3389/fpsyg.2020.00383 PMC7076912

[84] Thomas, G. & A. Dowker . 2000. Mathematics anxiety and related factors in young children. In British Psychological Society Developmental Section Conference , Bristol, UK.

[85] Carey, E. , A. Devine , F. Hill & F. Szűcs . 2017. Differentiating anxiety forms and their role in academic performance from primary to secondary school. PloS One 12: 1–20. 10.1371/journal.pone.0174418 PMC5370099 28350857

[86] Mammarella, I.C. , E. Donolato , S. Caviola & D. Giofrè . 2018. Anxiety profiles and protective factors: a latent profile analysis in children. Pers. Individ. Differ. 124: 201–208.

[87] Ramirez, G. , H. Chang , E.A. Maloney , *et al*. 2016. On the relationship between math anxiety and math achievement in early elementary school: the role of problem solving strategies. J. Exp. Child Psychol. 141: 83–100. 26342473 10.1016/j.jecp.2015.07.014

[88] Wu, S. , H. Amin , M. Barth , *et al*. 2012. Math anxiety in second and third graders and its relation to mathematics achievement. Front. Psychol. 3: 1–11. 22701105 10.3389/fpsyg.2012.00162 PMC3369194

[89] Wu, S.S. , E.G., Willcutt , E. Escovar & V. Menon . 2014. Mathematics achievement and anxiety and their relation to internalizing and externalizing behaviors. J. Learn. Disabil. 47: 503–514. 23313869 10.1177/0022219412473154 PMC3883980

[90] White, S.W. , D. Oswald , T. Ollendick & L. Scahill . 2009. Anxiety in children and adolescents with autism spectrum disorders. Clin. Psychol. Rev. 29: 216–229. 19223098 10.1016/j.cpr.2009.01.003 PMC2692135

[91] Tripp, G. , E.A. Schaughency & B. Clarke . 2006. Parent and teacher rating scales in the evaluation of attention‐deficit hyperactivity disorder: contribution to diagnosis and differential diagnosis in clinically referred children. J. Dev. Behav. Pediatr. 27: 209–218. 16775518 10.1097/00004703-200606000-00006

[92] Friso‐Van den Bos, I. , S.H. Van der Ven , E.H. Kroesbergen & J.E. Van Luit . 2013. Working memory and mathematics in primary school children: a meta‐analysis. Educ. Res. Rev. 10: 29–44.

[93] Jarvis, H.L. & S.E. Gathercole . 2003. Verbal and non‐verbal working memory and achievements on national curriculum tests at 11 and 14 years of age. Educ. Child Psychol. 20: 123–140.

[94] Reuhkala, M. 2001. Mathematical skills in ninth‐graders: relationship with visuo‐spatial abilities and working memory. Educ. Psychol. 21: 387–399.

[95] Peng, P. , J. Namkung , M. Barnes & C. Sun . 2016. A meta‐analysis of mathematics and working memory: moderating effects of working memory domain, type of mathematics skill, and sample characteristics. J. Educ. Psychol. 108: 455–473.

[96] Donolato, E. , D. Giofrè & I.C. Mammarella . 2019. Working memory, negative affect and personal assets: how do they relate to mathematics and reading literacy? PloS One 14: 1–17. 10.1371/journal.pone.0218921 PMC6597163 31246987

[97] Donolato, E. , E. Toffalini , D. Giofrè , *et al*. 2020. Going beyond mathematics anxiety in primary and middle school students: the role of ego‐resiliency in mathematics? Mind Brain Educ. 14: 255–266.

[98] Ashcraft, M.H. & A.M. Moore . 2009. Mathematics anxiety and the affective drop in performance. J. Psychoeduc. Assess. 27: 197–205.

[99] Passolunghi, M.C. , C. De Vita & S. Pellizzoni . 2020. Math anxiety and math achievement: the effects of emotional and math strategy training. Dev. Sci. 22: 1–11. 10.1111/desc.12964 32159906

